# Supplementary material for: The T4bSS of Legionella features a two-step secretion pathway with an inner membrane intermediate for secretion of transmembrane effectors
Source: bioRxiv. 2024 Mar 14:2024.03.14.584949. Preprint. [Version 1] doi: 10.1101/2024.03.14.584949 (PMC10980071; doi:10.1101/2024.03.14.584949)
Supplement: 1 [file NIHPP2024.03.14.584949v1-supplement-1.pdf]

bioRxiv preprint doi: https://doi.org/10.1101/2024.03.14.584949; this version posted March 14, 2024. The copyright holder for this preprint (which was not certified by peer review) is the author/funder, who has granted bioRxiv a license to display the preprint in perpetuity. It is made available under aCC-BY 4.0 International license.

**Supplemental Information**

**S1 Table 3line file of the DeepTMHMM prediction of the 82 previously reported TMD-effectors**

**S2 Table gff3 file of the DeepTMHMM prediction of the 82 previously reported TMD-effectors**

**S3 Table DeepTMHMM prediction of the 82 previously reported TMD-effectors**

**S4 Table Primers, Plasmids and Strains**

**S5 Table Statistics, p-values**

**S1 Fig. Distribution of T4bSS components across the membrane-fractionating sucrose gradient**

bioRxiv preprint doi: https://doi.org/10.1101/2024.03.14.584949; this version posted March 14, 2024. The copyright holder for this preprint (which was not certified by peer review) is the author/funder, who has granted bioRxiv a license to display the preprint in perpetuity. It is made available under aCC-BY 4.0 International license.

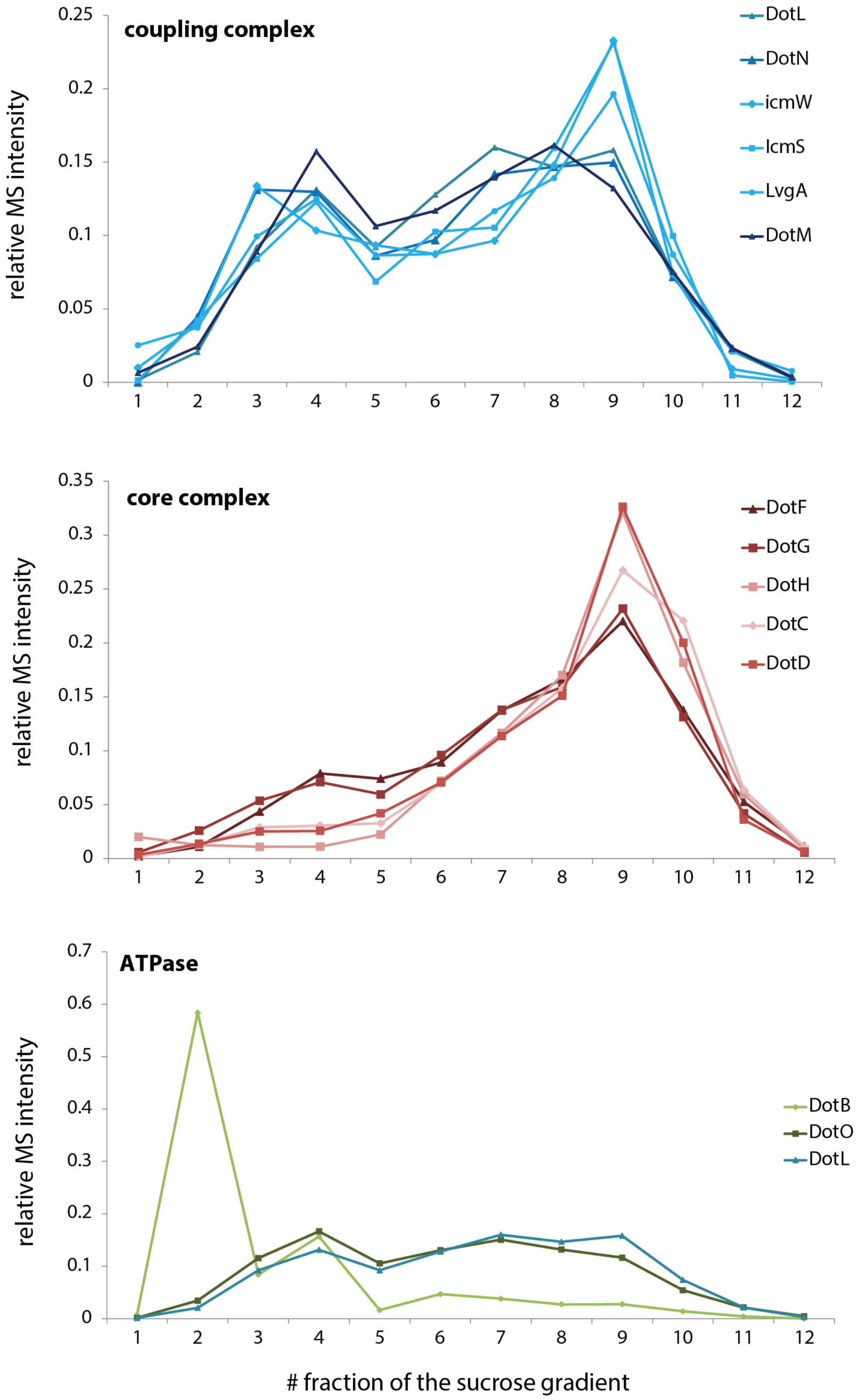


**S1 Fig. Distribution of T4bSS components across the membrane-fractionating sucrose gradient**

The relative abundance of the indicated T4bSS components in 12 fractions of a membrane-fractionating sucrose gradient as analyzed by mass spectrometry. The coupling complex components as well as DotO and DotL follow the distribution of known inner membrane proteins (see Fig. 1A). DotB follows the distribution of soluble proteins and the core complex components follow the distribution of outer membrane proteins. The equilibration of the core complex in the outer membrane fractions may result from the high density of vesicles that contain the large core complex.
